# Interplay between Toxin Transport and Flotillin Localization

**DOI:** 10.1371/journal.pone.0008844

**Published:** 2010-01-22

**Authors:** Sascha Pust, Anne Berit Dyve, Maria L. Torgersen, Bo van Deurs, Kirsten Sandvig

**Affiliations:** 1 Centre for Cancer Biomedicine, University of Oslo, Oslo, Norway; 2 Department of Biochemistry, Institute for Cancer Research, The Norwegian Radium Hospital, Oslo University Hospital, Oslo, Norway; 3 Structural Cell Biology Unit, Department of Cellular and Molecular Medicine, The Panum Institute, University of Copenhagen, Copenhagen, Denmark; University of Geveva, Switzerland

## Abstract

The flotillin proteins are localized in lipid domains at the plasma membrane as well as in intracellular compartments. In the present study, we examined the importance of flotillin-1 and flotillin-2 for the uptake and transport of the bacterial Shiga toxin (Stx) and the plant toxin ricin and we investigated whether toxin binding and uptake were associated with flotillin relocalization. We observed a toxin-induced redistribution of the flotillins, which seemed to be regulated in a p38-dependent manner. Our experiments provide no evidence for a changed endocytic uptake of Stx or ricin in cells silenced for flotillin-1 or -2. However, the Golgi-dependent sulfation of both toxins was significantly reduced in flotillin knockdown cells. Interestingly, when the transport of ricin to the ER was investigated, we obtained an increased mannosylation of ricin in flotillin-1 and flotillin-2 knockdown cells. The toxicity of both toxins was twofold increased in flotillin-depleted cells. Since BFA (Brefeldin A) inhibits the toxicity even in flotillin knockdown cells, the retrograde toxin transport is apparently still Golgi-dependent. Thus, flotillin proteins regulate and facilitate the retrograde transport of Stx and ricin.

## Introduction

The flotillin protein family consists of the two highly conserved molecules flotillin-1 and -2, which are ubiquitously expressed. Originally, these proteins were reported to be upregulated during axon regeneration [1], [2], resulting in the alternative nomenclature reggie-1 (flotillin-2) and reggie-2 (flotillin-1). It has been reported that after oligomerization the flotillins are associated with clusters at the cell membrane [3]–[5]. The clusters (50–100 nm in size) seem to be different from caveolae, as it has convincingly been demonstrated that flotillins do not colocalize with caveolin [5], [6]. These non-caveolar rafts may act as scaffolding platforms to assemble different protein complexes [1], [5], [7], [8]. The localization of the flotillins is highly cell type dependent. In epithelial cells flotillin-1 and -2 are mainly localized at the plasma membrane and in endosomal/lysosomal compartments [3], [5], [9]. It has also been reported that flotillins are localized to the Golgi apparatus [10]. Moreover, experiments with flotillin mutants indicate a Golgi-dependent transport of flotillin-2 and a similar trafficking for flotillin-1 [11].

Over the last decade it has been discovered that flotillins are involved in a variety of cellular processes, including cell-matrix adhesion, phagocytosis, exocytosis, and several signaling pathways [3], [12]–[15]. In recent publications it has been reported that flotillins are crucial for the uptake of cholera toxin, and flotillins have been proposed to define a clathrin-independent endocytic pathway [11], [16], [17]. However, apart from their role in insulin signaling, where flotillin-2 forms a complex with CAP and Cbl, the underlying molecular mechanisms are poorly understood [12].

The Shiga toxin (Stx) is a member of the bacterial AB_5_ toxins, consisting of an enzymatically active A-moiety that is noncovalently associated with a pentameric B-moiety, which mediates the binding to the glycolipid Gb3 at the cell surface and the subsequent endocytic uptake [18], [19]. It has been demonstrated recently that the composition of the glycophingolipids in the cellular membrane is crucial for the uptake of Stx [20]. Moreover, Stx itself induces Gb3 clustering, lipid reorganization and changes in the membrane curvature [21]–[23]. After endocytosis via clathrin-dependent and partially clathrin-independent mechanisms [23], [24], Stx is retrogradely transported from endosomal structures to the Golgi and further to the endoplasmic reticulum [25]. From the ER, the enzymatically active A1 subunit is translocated to the cytoplasm and inhibits protein synthesis by modification of 28S RNA of ribosomes. Interestingly, Stx is also able to induce signaling that activates its own uptake and trafficking to the Golgi apparatus [26]–[30].

The plant toxin ricin, isolated from *Ricinus communis*, consists of the two polypeptide chains A and B, which are linked via a disulfide bridge [24]. Also in ricin the A-chain represents the enzymatically active component, leading to modification and inactivation of 28S ribosomal RNA [31]. The binding to glycolipids or glycoproteins with terminal galactose and the subsequent endocytic uptake is mediated via the B-chain. Similarly as for Stx, the importance of cholesterol in ricin transport has been described [32]–[34]. Both toxins are partly recycled from endosomal compartments to the cell surface, partly transported to the Golgi apparatus and the ER, and partly degraded in lysosomes [19], [35]. From the ER, ricin A is translocated into the cytoplasm to inhibit protein biosynthesis, triggering apoptotic events [36], [37].

In the present study we investigated the role of flotillins in endocytosis and retrograde transport of Shiga toxin and ricin and we analyzed to which extent the toxins affect the localization of flotillins. Our experiments indicate that Stx and ricin induce a p38 MAPK-dependent redistribution of both flotillins. In contrast to the results reported for cholera toxin [17], we found no reduced endocytic uptake of Stx or ricin in cells silenced for flotillin-1 or -2. However, we obtained a significant reduction in the sulfation (a Golgi-dependent modification) of both toxins after flotillin knockdown especially by using flotillin-1 siRNA oligos, suggesting that the flotillin proteins are important for Golgi transport. Despite that, the trafficking of ricin to the ER seemed to be increased after depletion of flotillins, since mannosylation (an ER-dependent modification) as well as toxicity of ricin was increased. Under similar conditions also the toxicity of Stx was increased. Thus, knockdown of flotillins facilitate the transport of Stx and ricin to the cytosol and additionally the localization of flotillins is affected by the toxins.

## Results

### Stx and Ricin Partially Colocalize with Flotillin-1/-2 and Induce a Change in the Cellular Localization of Flotillins

**Figure 1:**
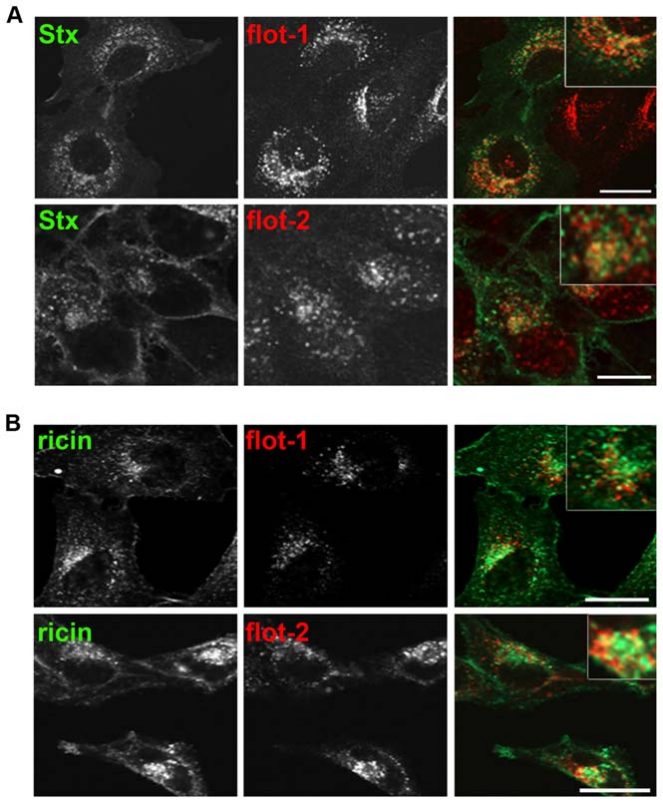
Stx and ricin are colocalizing with flotillin-1 and -2. (A) HeLa cells were seeded on glass coverslips and after 24 h cells were treated for 30 min with 200 ng/ml Stx, fixed, permeabilized, and labeled for Stx (green) and flotillin-1 or -2 antibodies (red). Pictures in (A) and (B) were analyzed by using Zeiss LSM Image Browser. Bars: 20 µm. (B) As described for (A) but HeLa cells were treated for 2 h with 1 µg/ml of a Cy-2 labeled ricin sulf-1 and stained for flotillin-1 or -2. Scale bars: 20 µm.

First, we investigated by confocal microscopy the localization of Stx, ricin and flotillin. HeLa cells, incubated for 30 min with Stx, showed some colocalization of Stx with flotillin-1 in perinuclear structures (Figure 1A). Also, flotillin-2 was found to colocalize with Stx to a lower extent. Similar results were obtained in HEp-2 cells and when HeLa or HEp-2 cells were treated with the Stx B subunit containing two sulfation-sites (StxB sulf-2; not shown). As it is known that the mechanisms regulating endocytosis and intracellular transport are different for ricin and Stx [27], [28], we also checked for a potential colocalization between the flotillins and ricin. For the visualization of ricin by confocal microscopy, ricin sulf-1 was fluorescently labeled with Cy2. Comparable to the results obtained for Stx, ricin was after 2 h partially colocalized with flotillin-1 and flotillin-2 in perinuclear structures both in HeLa and HEp-2 cells (Figure 1B).

To get a more detailed picture of the localization of flotillins on endosomal structures, we took advantage of a HeLa cell line expressing a constitutively active Rab5 mutant (Q79L) in an inducible manner. This active Rab5 leads to increased endosomal membrane fusion, resulting in the formation of enlarged endosomes, which can be visualized by confocal microscopy [38], [39]. We analyzed the endosomal localization of Stx as well as the localization of flotillin-1 and -2. Stx was partially localized to the same endosomal structures as the flotillins (Figure S1). However, Stx was also found on enlarged endosomes that were negative for flotillin staining. On the other hand, flotillins could be found on endosomal structures without any staining for Stx or ricin. Furthermore, confocal microscopy of Stx and flotillin-1 and -2 in HeLa cells expressing Rab5 Q79L indicates that, although flotillins and Stx can be found in the same endosomes, they are partially localized to different membrane domains.

**Figure 2:**
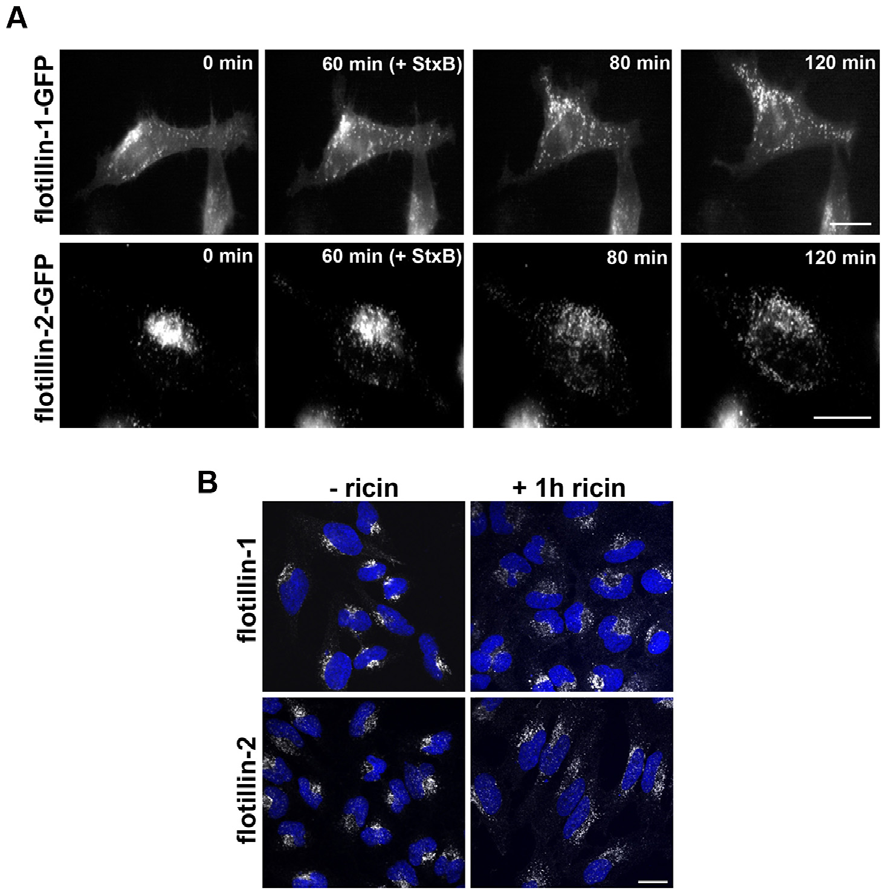
Treatment of Stx or ricin leads to redistribution of flotillin-1 and -2. (A) HeLa cells were transfected with flotillin-1-GFP or flotillin-2-GFP. The localization of the GFP-labeled proteins was visualized with a BioStation IM for 60 min without toxin. After 60 min 2 µg/ml of StxB sulf-2 was added and the relocalization was analyzed (pictures were taken every 3 min for 2 h in total. Scale Bars: 20 µm). (B) HeLa control cells or ricin sulf-2 treated cells (1 h) were fixed and stained for flotillin-1 or -2 and nuclei (blue) and analyzed by confocal microscopy. Scale bars: 20 µm.

We next analyzed whether binding and internalization of Stx and ricin had an effect on the localization of flotillins. To this end, we examined by confocal laser-microscopy or by time-lapse microscopy the cellular distribution of endogenous or GFP-labeled flotillin-1 and -2 in cells treated with Shiga toxin or ricin. For time-lapse microscopy, HeLa cells were transfected with flotillin-1- or flotillin-2-GFP constructs for 24 h. Single cells were visualized for 2 h in total. In untreated HeLa cells, the labeled flotillins localized to some extent to the plasma membrane, whereas, the majority of the flotillins was found in a perinuclear area (Figure 2). The localization of both flotillins was analyzed by time-lapse microscopy with GFP-tagged flotillins in HeLa cells (Figure 2A). Untreated cells were observed for 60 min and flotillins were mainly localized in perinuclear areas (Fig. 2A, 0 min and 60 min). When StxB sulf-2 was added after 60 min, both flotillins started to redistribute within 20–60 min to vesicles with widespread cytoplasmic localization (Fig. 2A, 80 min and 120 min). Similar effects were observed in HeLa cells after 1 h of ricin sulf-1 treatment (Figure 2B). This redistribution could be seen also for endogenous flotillins in HeLa and HEp-2 cells treated with ricin holotoxin, StxB, or Shiga holotoxin (not shown).

**Figure 3:**
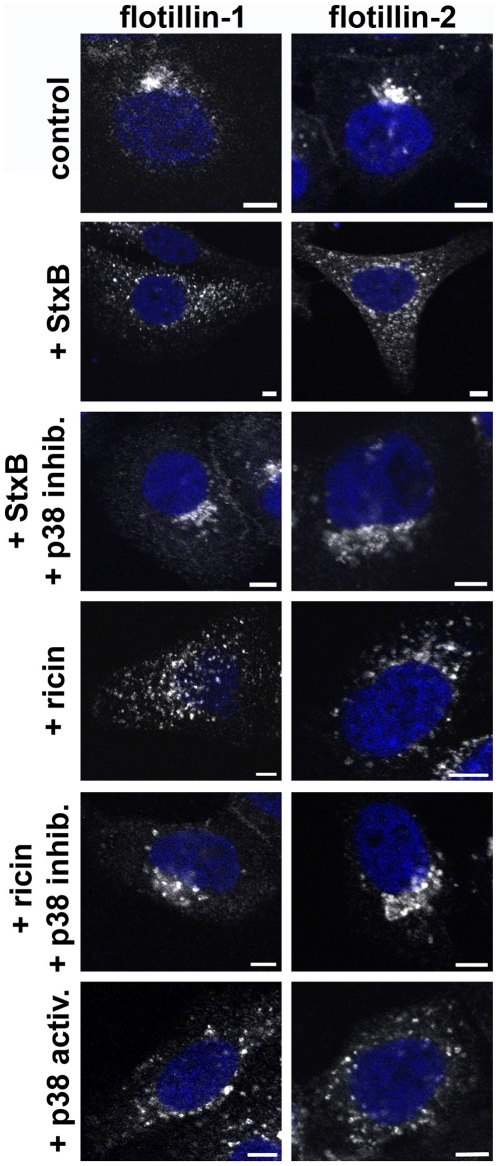
Toxin-induced flotillin redistribution is mediated by p38 MAPK. HeLa cells were incubated for 30 min with StxB sulf-2 or ricin sulf-1. Alternatively, cells were pretreated for 30 min with 20 µM of SB203580 (p38 MAPK inhibitor) and subsequently treated for 30 min with StxB sulf-2 or ricin sulf-2, treated for 20 min only with 10 µM of anisomycin (p38 MAPK activator) or left untreated (control). Cells were subsequently fixed and stained for flotillin-1 or -2. Bars: 5 µm.

Among a variety of other proteins, p38 has been reported to be activated by Stx and crucial for the endosome to Golgi transport of Stx [28], [40]. In order to investigate the mechanism behind the redistribution of flotillins, we tested the possible involvement of p38 MAP kinase. For this propose, we used SB203580, a chemical inhibitor of p38 MAP kinase (Figure 3). Pretreatment with SB203580 for 30 min inhibited the Stx and ricin induced redistribution of flotillins in HeLa cells. Interestingly, anisomycin, an activator of p38 MAP kinase, also induced a redistribution of flotillins both in HeLa and in HEp-2 cells.

In conclusion, the microscopy analysis indicates that the distribution of the flotillins is highly dynamic, affected by toxin treatment, and dependent on p38 MAPK.

### The Toxicity of Stx and Ricin Is Increased after Knockdown of the Flotillin-1 or Flotillin-2

**Figure 4:**
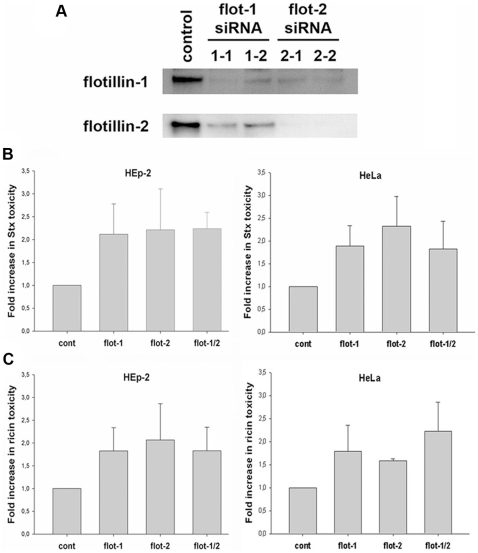
Flotillin-depletion leads to an increased toxicity of Shiga and ricin. (A) HeLa cells were transfected with either non-targeting siRNA (control) or two different siRNA oligos targeting flotillin-1 (1–1 and 1–2) or flotillin-2 (2–1 and 2–2) and incubated for 3 d. The amount of flotillin-1 and flotillin-2 was analyzed by Western blot with the indicated antibodies. (B+C) HEp-2 and HeLa cells were transfected with the corresponding siRNA oligos for 3 d. Subsequently, cells were treated for 30 min with leucine-free medium and increasing amounts of Stx or ricin were added, incubation was continued for 1.5 h (Stx) or 2 h (ricin). Protein biosynthesis was measured by [^3^H]leucine incorporation, and the increase in Stx or ricin toxicity was calculated at 50% inhibition of protein biosynthesis.

We then investigated the influence of flotillins on the toxicity of Stx or ricin. For that purpose, siRNA oligos were used to knockdown flotillin-1 or -2. The knockdown efficiency with two different siRNA oligos for flotillin-1 and -2 was determined by Western-blot analysis in HeLa and HEp-2 cells three days after transfection (Figure 4A). The knockdown efficiency for flotillins-1 and -2 was 75–95% in all performed experiments. Therefore, in the following results only data with one siRNA oligo pair are shown. As already described by other groups [4], [41], the knockdown of flotillin-2 led to a reduced protein level of flotillin-1, most likely due to protein destabilization and subsequent degradation. For that reason we are not fully able to distinguish between effects of flotillin-1 and -2.

To study the effect of flotillin depletion on toxicity of Stx and ricin, we analyzed the toxin-induced inhibition of protein biosynthesis. Knockdown of flotillin-1, flotillin-2 or both flotillins led to a significant increase in the Stx toxicity (Figure 4B) as well as in the ricin toxicity (Figure 4C). In HEp-2 cells the flotillin depletion led to a ∼2 fold increase in Stx and ricin toxicity compared to control cells, calculated at 50% inhibition of protein biosynthesis. The double knockdown with siRNA oligos for flotillin-1 and -2 did not lead to a significant additional increase in the toxicity compared to the single knockdowns.

To study by which mechanism the flotillins are affecting the toxicity of Stx and ricin, we started to investigate the endocytosis and retrograde transport of Stx and ricin in flotillin depleted cells.

### The Endocytosis of Stx and Ricin Is Independent of Flotillins

**Figure 5:**
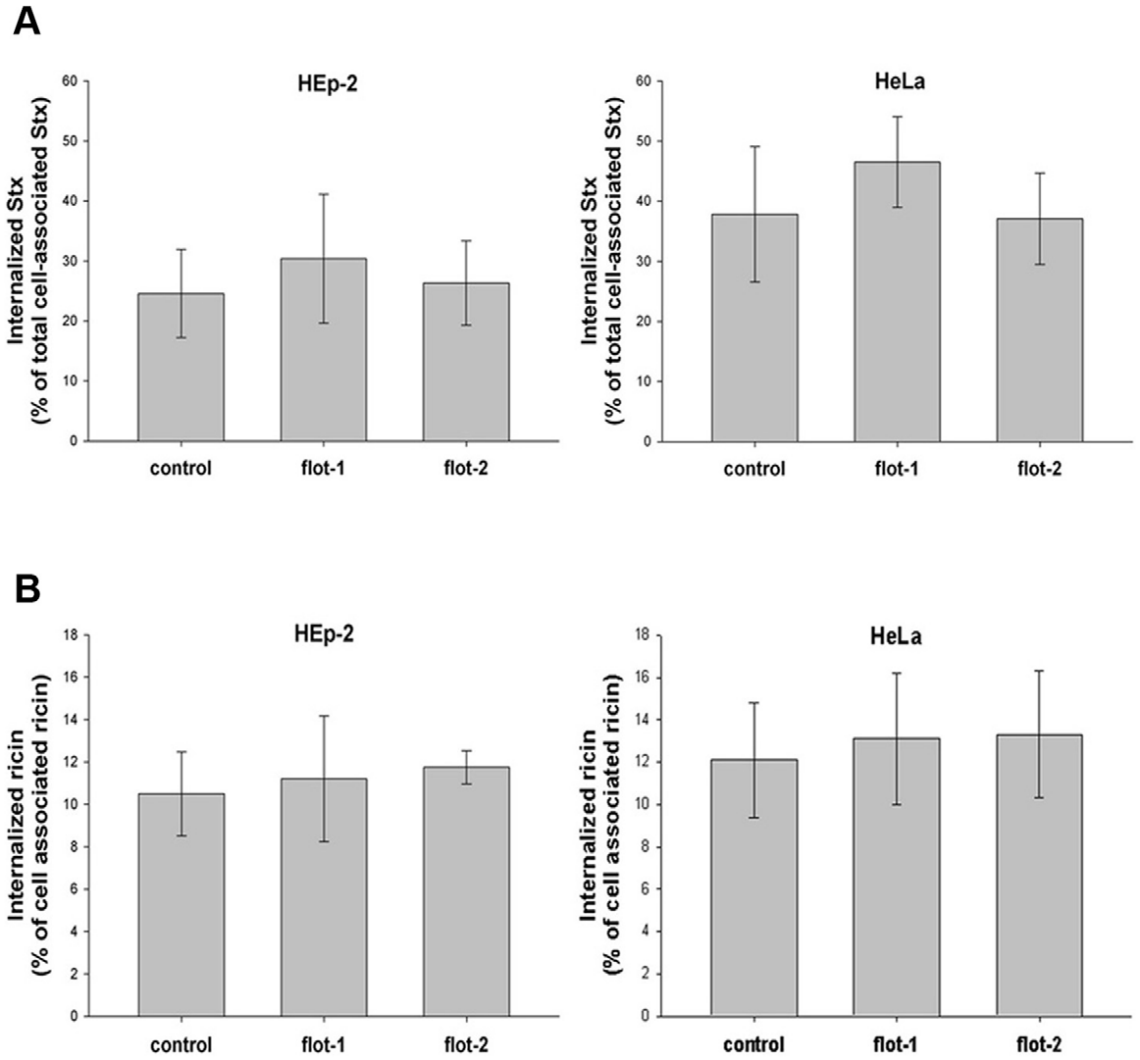
Endocytosis of ricin and Shiga toxin is independent of flotillins. (A) HeLa or HEp-2 cells were transfected with the corresponding siRNA oligos and incubated for 3 d before 0.33 nM of biotinylated Stx was added for 30 min at 37°C. Cells were treated with or without MESNa and internalized biotin-Stx was precipitated by streptavidin-coated beads and measured by electrochemiluminescence via a Ru(II)-tag labeled Stx antibody. (B) HEp-2 or HeLa cells were transfected as described in (A) and treated with ^125^I-ricin for 20 min at 37°C. To calculate the amount of internalized ricin, half of the samples were washed with 0.1 M lactose to remove membrane-bound toxin. The amount of internalized ricin was determined as the percentage of total cell associated toxin.

Lipid rafts have been found to play a role in the uptake and retrograde transport of protein toxins and flotillin proteins are frequently used as markers of these structures. Therefore, as the toxicity of Stx and ricin was significantly increased in flotillin-depleted cells, we decided to investigate the role of flotillins in the uptake and intracellular transport of Stx and ricin. Flotillin-depleted HEp-2 or HeLa cells were treated with biotin-labeled Stx and the binding and uptake efficiencies were measured after 30 min of incubation, as shown in Figure 5. Neither single knockdown in HEp-2 and HeLa cells nor double knockdown with flotillin-1 and flotillin-2 specific oligos (not shown) changed significantly binding (not shown) or the uptake of Shiga toxin (Figure 5A).

For quantification of ricin uptake, cells were incubated for 20 min with ^125^I-labeled ricin and the amounts of internalized and cell-associated toxin were determined (Figure 5B). Similarly to the results for Stx, depletion of flotillins did not change the binding or internalization of ricin.

As the increase in the toxicity of Stx and ricin is not due to an altered endocytosis of these toxins, we next studied the role of the flotillins in intracellular toxin transport.

### Knockdown of Flotillin Proteins Reduces the Sulfation of StxB and Ricin

**Figure 6:**
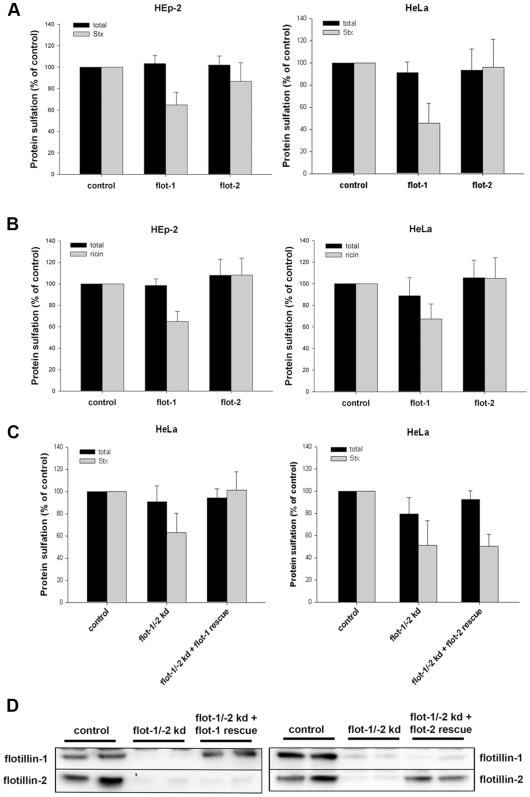
Knockdown of flotillin-1 leads to a decreased sulfation of Shiga toxin and ricin. (A) HEp-2 or HeLa cells were transfected with 25 nM of the indicated siRNA oligos and incubated for 3 d before they were treated with StxB sulf-2 for 1 h at 37°C in sulfate-free medium in presence of H_2_^35^SO_4_. Cells were lysed and StxB was immunoprecipitated, sulfated Stx was analyzed by SDS-PAGE and autoradiography. The amount of StxB sulfation and total protein sulfation was plotted + SD. (B) As described in (A), but cells were treated with ricin sulf-1 for 2 h instead of StxB sulf-2. (C) As described in (A), but cells were depleted for flotillin-1 and -2 (15 nM of siRNA oligos each) and transfected with siRNA resistant flotillin-1 or -2 for 24 h in total. (D) Representative Western blots of flotillin-1 and -2 levels in HeLa cells, used for experiments shown in (C).

Following the uptake of Stx or ricin, the toxins are transported from endosomes to the Golgi. Proteins, containing sulfation sites, are specifically sulfated in the trans-Golgi network (TGN) [42], [43]. To quantify the amount of Stx and ricin transported from endosomes towards the TGN, we used the B-moiety of Stx with a tandem sulfation site [44] at the C-terminus (StxB sulf-2) and a modified ricin A (ricinA sulf-1) with a tyrosine sulfation site [37] reconstituted with ricin B. HEp-2 or HeLa cells, depleted either for flotillin-1 or flotillin-2, were treated with StxB sulf-2 or ricin sulf-1 in the presence of radioactively labeled sulfate. As shown in Figure 6A, the knockdown of flotillin-1 led to a significant reduction in the StxB sulfation: ∼40% and 55% reduction in HEp-2 and HeLa cells, respectively. However, the Stx sulfation was only very slightly reduced after knockdown of flotillin-2 in Hep-2 cells (14%) and HeLa cells (4%). Similar results were obtained when we analyzed the amount of sulfated ricin after flotillin knockdown (Figure 6B). After knockdown with flotillin-1 specific siRNA oligos, the sulfation of ricin was reduced to 65% in Hep-2 cells and to 67% in HeLa cells compared to the control, whereas the knockdown of flotillin-2 had no significant effect on the ricin-sulfation. The double knockdown with flotillin-1 and flotillin-2 oligos decreased the ricin- and Stx-sulfation to the same level as observed after knockdown with flotillin-1 oligos. Control experiments showed that the total sulfation in the cell, which was measured for every condition, was either unchanged or only slightly affected after knockdown of flotillins.

In order to distinguish between the effect of flotillin-1 and flotillin-2 and to determine the specificity of the siRNA oligos we performed rescue experiments. HeLa cells were depleted for flotillin-1 and -2 and rescued either with siRNA resistant flotillin-1 or flotillin-2 (Fig. 6C). In cells rescued for flotillin-1, Stx sulfation was restored to control level, whereas, rescue with flotillin-2 did not alter Stx sulfation. Transfection with siRNA resistant flotillins led in flotillin-depleted cells to a specific expression of flotillin-1 or -2 at a level comparable to the expression level in control cells (Fig. 6D). These results indicate a specific role of flotillin-1 in the endosome-to-Golgi transport.

**Figure 7:**
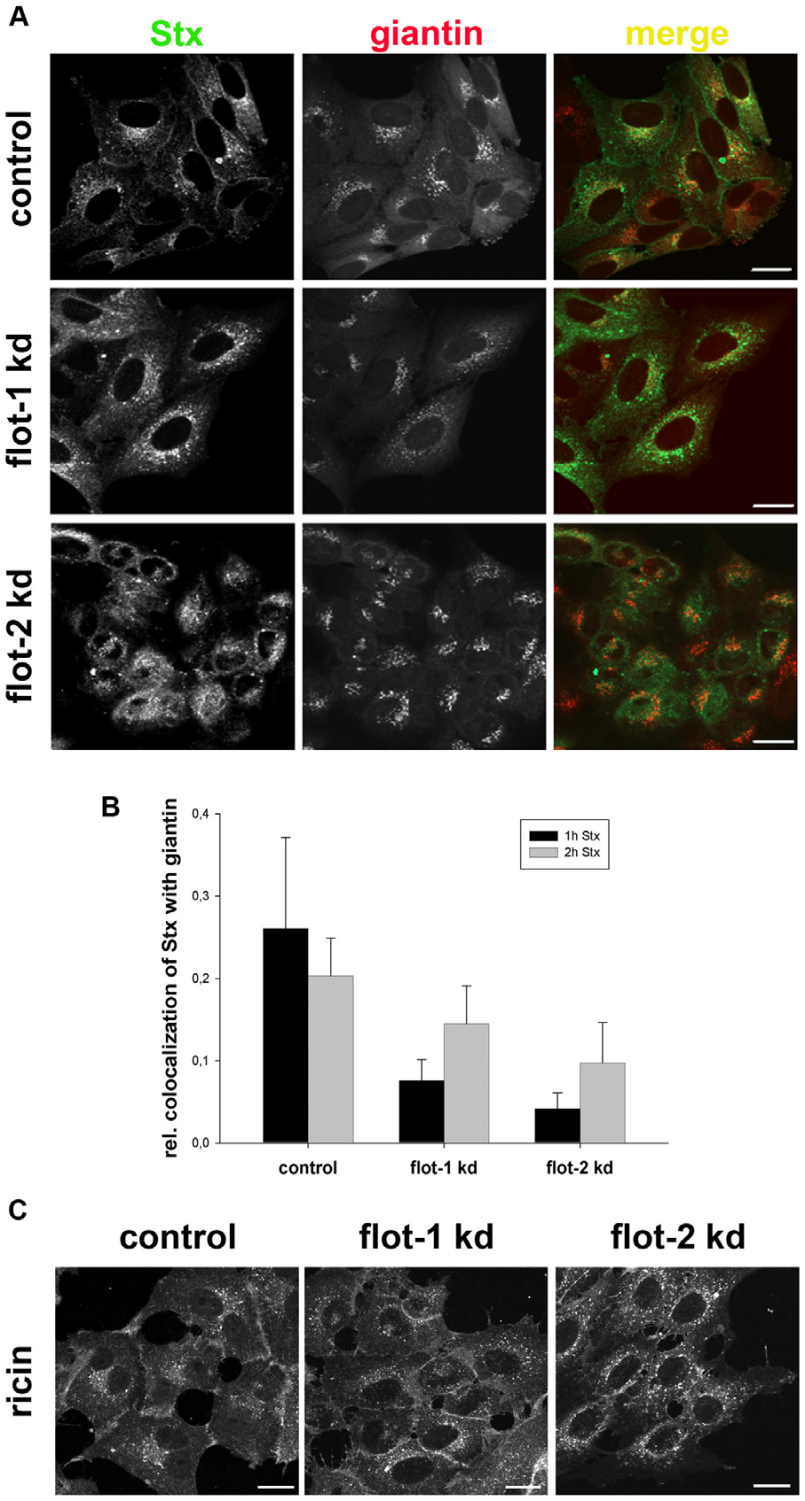
Depletion of flotillins changes the intracellular localization of Stx. (A) HEp-2 cells were transfected with 25 nM of the indicated siRNA oligos and incubated for 3 d before they were treated with 200 ng/ml of Stx for 1 h. Subsequently cells were fixed, permeabilized and stained for Stx and the Golgi-marker giantin. Bars: 20 µm. In parallel, the knockdown efficiency of flotillin-1 and -2 was determined by Western blot analysis (∼75%). (B) The relative colocalization of Stx and giantin after 1 h and 2 h treatment was quantified by ImageJ software. (C) HEp-2 cells were transfected with 25 nM of the indicated siRNAs and incubated for 3 d and subsequently treated with Cy2-labeled ricin sulf-1 for 1 h. Bars: 20 µM.

To get more information about the trafficking of Stx from endosomes to the Golgi we performed confocal microscopy studies. For this purpose, we analyzed the distribution of Stx and ricin in flotillin-depleted cells. Here, we observed a change in the localization of Stx in flotillin-depleted cells. The localization of Stx after 1 h incubation in knockdown HEp-2 cells was significantly changed from a perinuclear to a more dispersed localization (Figure 7A). This altered distribution was also quantified by analyzing the colocalization between Stx and the Golgi marker giantin (Figure 7B). Here, we measured a significant reduction in the colocalization of Stx with the Golgi. Similar results were obtained using HeLa cells. In contrast to Stx, the intracellular localization of ricin is more widespread, and there was no obvious change in flotillin depleted cells (Figure 7C). The possibility exists that knockdown of flotillins might affect the Golgi apparatus per se. However, no effect could be seen on the perinuclear localization of the Golgi marker, and also studies with flotillin-depleted cells revealed no detectable morphological changes at the EM level (data not shown).

### The Mannosylation of Ricin Is Increased after Knockdown of Flotillins

**Figure 8:**
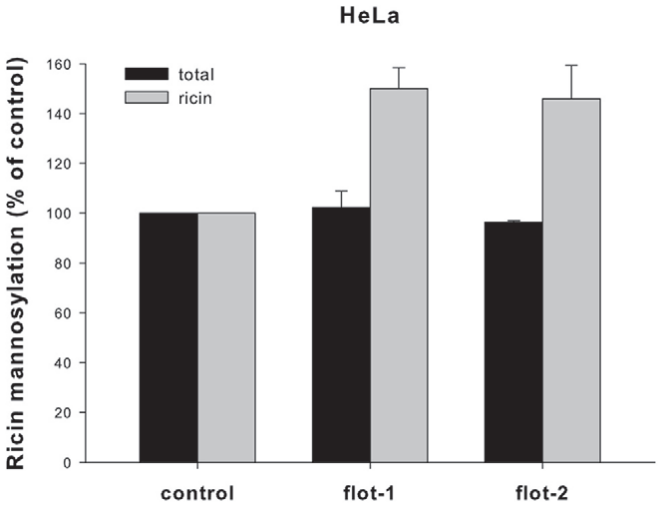
Ricin mannosylation is increased after flotillin-1 or -2 knockdown. HeLa cells were transfected with the indicated siRNA oligos and incubated for 3 d before they were treated with ricin sulf-2 for 3 h in presence of [^3^H]mannose. Subsequently, cells were lysed and the amount of incorporated [^3^H]mannose was analyzed by SDS-PAGE and autoradiography. The total protein mannosylation and ricin sulf-2 mannosylation was quantified and plotted + SD.

After the transport of the toxins into the TGN, the next step in retrograde transport is the transport through the Golgi and to the ER. To quantify this transport step, we took advantage of a modified ricin protein (ricin sulf-2), which contains a tyrosine sulfation site and three partially overlapping N-glycosylation sites C-terminally of the A-chain [37]. Since it is known that addition of mannose-containing N-linked oligosaccharides occurs in the ER, we directly measured the glycosylation of the ricin molecules using radioactively labeled mannose. The signals obtained in HEp-2 cells were too weak to analyze and therefore the experiments were performed in HeLa cells only (Figure 8). The total amount of cellular incorporated [^3^H]mannose was not affected by knockdown of flotillin-1 or flotillin-2. However, the depletion of flotillins led to a significantly increased mannosylation of ricin (∼150% compared to the control), indicating a specific influence of flotillin-1 and/or flotillin-2 on the transport of ricin sulf-2 to the ER.

**Figure 9:**
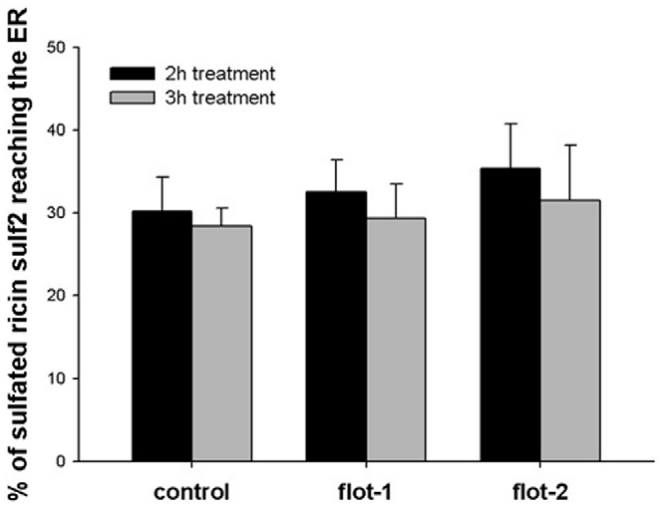
The amount of sulfated ricin sulf-2 reaching the ER is unchanged in flotillin depleted cells. HeLa cells were transfected for 3 d with 25 nM of the indicated siRNA oligos before treated with ricin sulf-2 for 2 h or 3 h at 37°C in presence of [^35^S]. Cells were lysed and ricin was immunoprecipitated. Subsequently, sulfated ricin was analyzed by SDS-PAGE and autoradiography. The amount of sulfated and glycosylated ricin relative to total sulfated ricin was quantified and plotted + SD.

As knockdown of flotillins seems to reduce toxin transport to the Golgi and simultaneously increase transport to the ER, we performed experiments to determine whether the toxins might be retrogradely transported in a Golgi-independent manner. When ricin sulf-2 is used in sulfation assays (2 h and 3 h of incubation), two bands can be visualized by autoradiography, representing sulfated and sulfated + glycosylated ricin [45], [46]. Therefore, it can be used to determine the fraction of ricin that has been sulfated in the TGN and subsequently transported to the ER, where it is mannosylated. Interestingly, knockdown of flotillins did not change the fraction of sulfated ricin that was also glycosylated (after 2 h or 3 h), i.e. transported to the ER (Figure 9). Moreover, when the transport of ricin to the ER was analyzed by incorporation of [^3^H]mannose, disruption of the Golgi apparatus by BFA led to a complete inhibition of this transport in both control and knockdown cells (Figure S2).

In conclusion, since the overall transport of ricin to the ER seems to be increased after flotillin knockdown (increased glycosylation and toxicity), some ricin molecules may circumvent the sulfotransferase-containing TGN. The toxin transport is apparently still Golgi-dependent, as it is BFA-sensitive.

## Discussion

The present study demonstrates a role for the flotillins in intracellular transport of Shiga toxin and ricin. Another important result is the Stx- and ricin-induced change of the flotillin distribution, which seems to be dependent on p38 MAPK. An inhibitor of this enzyme, SB203580, prevents the toxin-induced flotillin redistribution, indicating a role for p38 MAPK in flotillin localization. This is also supported by the fact that treatment with anisomycin, an activator of p38 MAPK, led to a change in the localization of flotillins in the absence of toxins. A Stx-induced activation a p38 MAPK has been described before [28], but no activation was found for ricin. However, the basal level of p38 might be sufficient for ricin-induced redistribution of flotillin. Interestingly, in a recent publication a flotillin-mediated Gq-induced p38 activation was discussed [47], indicating that the flotillins themselves are involved in the regulation of p38 and might form a complex that could affect their localization. This would be in analogy to our recent observation that p38 is important for the localization of arrestins [40]. Further experiments are needed to verify the specific role of p38 MAPK and to clarify whether other proteins are involved in the regulation of the flotillin distribution and how flotillin redistribution is temporally and spatially synchronized with Stx- and ricin transport.

No significant effect on the uptake of Stx after knockdown of flotillins was observed after 30 min, and when we analyzed the uptake in a time-dependent manner starting from 15 min up to 120 min (data not shown) still no difference could be observed in flotillin-depleted cells. Similarly, no effect was found on the uptake of ricin. However, a possible effect of flotillin-depletion on raft-mediated endocytosis of the toxins might be compensated by upregulation of other endocytic pathways.

The data obtained from sulfation experiments showed a reduced TGN-directed transport of Stx and ricin in flotillin-depleted cells. This effect was restricted to flotillin-1 oligos. However, in HEp-2 cells we also obtained a slight reduction in the Stx transport after knockdown of flotillin-2, which might be due to the fact that knockdown of flotillin-2 also affects the flotillin-1 level via destabilization and degradation of the protein [4]. In HeLa cells, no influence of flotillin-2 knockdown on Stx sulfation could be observed. For ricin transport to the Golgi apparatus, knockdown with flotillin-2 oligos had no effect, independently of the cell line. This finding is in line with previous studies, which have shown that it is much more difficult to affect ricin than Stx transport, maybe due to its ability to bind to a large variety of receptors. By using siRNA resistant flotillin-1 or -2 we were able to distinguish between specific effects of flotillin-1 or flotillin-2. The data demonstrate that flotillin-1, but not flotillin-2, is involved in the retrograde TGN-directed toxin transport. Interestingly, the means of membrane association for flotillin-1 and -2 are different. The membrane association of flotillin-1 is mediated by palmitoylation [48], which is a reversible modification. In addition to palmitoylation, flotillin-2 is irreversibly myristoylated [3]. As it is known that palmitoylation is a dynamic modification [49] that is involved in many cellular processes, such as trafficking and signaling [50], [51], it is likely that a change in the status of palmitoylation of flotillin-1 and -2 directly regulates their subcellular localization. With respect to different lipid-modification sites, flotillin-1 and flotillin-2 might therefore localize differently to the Golgi apparatus or associated protein complexes or vesicles. Thus, in flotillin-depleted cells the toxins might enter not only the TGN but also other, sulfotransferase-free, Golgi compartments, leading to a decreased sulfation.

In our knockdown experiments we were able to reduce the flotillin level to ≤25% compared to endogenous levels, independently of the siRNA oligonucleotides used. We cannot exclude that the flotillin levels after knockdown with the flotillin-2 oligos could be sufficient for toxin transport.

The decreased toxin transport to the Golgi and simultaneously increased ER-transport, raised the question of a Golgi-independent trafficking in flotillin-depleted cells. For cholera toxin and ricin a Golgi-independent toxin transport to the ER has been described [45], [52]. However, flotillin-depleted cells pretreated with BFA, which disrupts the TGN/Golgi, were insensitive to Stx and ricin treatment and no transport of ricin to the ER could be observed, suggesting a Golgi-dependent trafficking.

One possibility is that flotillin knockdown led to an accelerated trafficking through the Golgi. However, there was no significant difference in the fraction of sulfated ricin reaching the ER in control and flotillin-depleted cells. It is also conceivable that the flotillins are stabilizing the Golgi membrane and associated SNAREs, Rabs or sorting nexins and a knockdown could then lead to an altered vesicular trafficking. It is known that coassembly of flotillins induces membrane curvature [16], and membrane curvature itself is a driving force in Golgi organization [53]. Thus, flotillin knockdown might somehow lead to a change in the Golgi organization allowing incoming toxin-containing vesicles to fuse with other cisternae than the TGN. However, in flotillin knockdown cells no obvious morphological changes of the Golgi apparatus were observed in EM studies (data not shown). Finally, we can not rule out the possibility that the knockdown with flotillin-1 oligos but not with flotillin-2 oligos leads to a change in the localization of the sulfotransferase, which is responsible for the sulfation of Stx and ricin. However, we never observed a change in the total protein sulfation after flotillin knockdown in our experiments, suggesting that the activity of the sulfotransferase enzyme was not affected in general. In a recent publication it has been demonstrated that the trafficking of flotillins occurs in a Golgi-dependent manner [11]. This finding is in agreement with our data, suggesting a flotillin dependent Golgi-recruitment of retrogradely transported vesicles containing Stx and ricin. It will be interesting to know whether the flotillins play a general role in the retrograde transport of other toxins and cellular proteins.

The ricin transport to the ER, measured as increased glycosylation, was ∼50% increased after depletion of flotillins. This is apparently not a result of an increased enzyme activity of the corresponding glycosyltransferase, as the total protein mannosylation was not affected. Importantly, it supports the idea that the observed increase in toxicity is due to increased transport to the ER. As the data obtained in sulfation- and toxicity-assays were comparable for ricin and Stx, a similar effect on the transport of Stx to the ER is very likely.

In summary, we were able to show for the first time that flotillins are involved in the retrograde transport of Stx and ricin. In particular, our data suggest that retrograde toxin transport is regulated by flotillins. Furthermore, we provide data for a toxin induced and p38 MAPK-dependent distribution of the flotillins.

## Materials and Methods

### Materials

Hepes, bovine serum albumin, MESNa (mercaptoethanesulfonic acid), *n*-octylglucopyranoside, rabbit anti-ricin antibody, and rabbit anti-flotillin-2 antibody were purchased from Sigma-Aldrich (St. Louis, MO, USA). H_2_^35^SO_4_ and [^3^H]leucine were from Hartman Analytic (Braunschweig, Germany), D-[2-^3^H(N)]-mannose and Na^125^I were purchased from Perkin Elmer (Boston, MA, USA). Brefeldin A was from Epicentre Biotechnologies (Madison, MI, USA). Mouse anti-Stx antibodies (13C4 and 3C10) were from Toxin Technology (Sarasota, FL, USA), the monoclonal anti-flotillin antibodies were from BD Bioscience (San Jose, CA), the rabbit anti-flotillin-1 antibody was a gift from Dr. G. van der Goot (EPFL, Lausanne, Switzerland) and rabbit anti-EEA-1 was from Cell Signaling Technology (Danvers, MA, USA). Shiga toxin (Stx) was provided by Dr. J. V. Kozlov (Academy of Sciences of Russia, Moscow, Russia) and by Dr. J. E. Brown (U.S. Army Medical Research Institute of Infectious Diseases, Fort Detrick, MD, USA). The plasmid encoding StxB-sulf2 was a kind gift from Dr. B. Goud (Institute Curie, Paris, France). The protein concentrations in the lysates were determined by the BCA protein assay (Pierce) with the use of bovine serum albumin as the standard. Ricin sulf-1 was fluorescently labeled with Cy2 bis-Reactive Dye Pack (GE Healthcare Biosciences, Uppsala, Sweden), and purified with Fluorescent Dye Removal Columns purchased from Thermo Scientific (Rockford, Il, USA), according to the manufacturer's procedure.

### Cell Culture and Transfection

HeLa and HEp-2 cells were grown under 5% CO_2_ in Dulbecco's modified Eagle's medium (Invitrogen, Carlsbad, CA, USA) supplemented with 10% fetal calf serum, 100 units/ml penicillin, 100 µg/ml streptomycin, and L-glutamine at 2 mM. HeLa rab5 Q79L cells were grown as described elsewhere [54]. For sulfation experiments, the cells were seeded out in 6-well plates at a density of 3×10^4^ cells/well, 24 h before the experiments. For endocytosis and toxicity experiments the cells were seeded out in 24-well plates at a density of 8×10^3^ cells/well and grown for 24 h before the experiments were started. Two different siRNAs targeting non-overlapping parts of the mRNA sequence were used for flotillin-1 and -2. The sequences of the flotillin-1 targeting constructs were GCAGAGAAGUCCCAACUAAUU and UGGCCAAGGCACAGAGAGA (Flot 1–1 and 1–2, respectively), and of the flotillin-2 targeting construct GAGGUUGUGCAGCGCAAUU and GGAUGAAGCUCAAGGCAGA (Flot 2–1 and 2–2, respectively). To reduce unspecific off-target effects, the constructs were ordered as ON-TARGETplus oligos from Dharmacon RNAi Technologies (Chicago, IL, USA). For siRNA transfection the cells were seeded out without antibiotics, grown for 24 h, and transfected by using DharmaFECT™1 from Dharmacon RNAi Technologies according to the manufacturer's procedure. After 4 h of transfection, the medium was changed to complete growth medium containing serum and antibiotics, and the cells were grown for 3 days before experiments were started. For time-lapse microscopy HeLa cells were transfected with the flotillin-1-GFP or flotillin-2-GFP constructs for 24 h by using FuGene6 (Roche Diagnostics, Mannheim, Germany) as described by the manufacturer. For rescue experiments siRNA resistant flotillin-1 and -2 (DNA 2.0, Menlo Park, CA, USA) were cloned into the mammalian expression vector pcDNA3 (Invitrogen). Hela cells were seeded and transfected as described above and 2 days after transfection with siRNA oligos cells were transfected for 24 h with 1 µg of siRNA resistant flotillin-1 or -2 or empty pcDNA3 vector by using FuGene6.

### Measurement of Stx and Ricin Endocytosis

The endocytosis of Stx was measured and quantified by an electro-chemiluminescent detection instrument (BioVeris Corporation, Gaithersburgh, MD, USA) as described in detail previously [27].

Binding and endocytosis of ricin was measured after 20 min at 37°C. The amount of internalized ^125^I-labeled toxin was measured after incubating the cells with a 0.1 M lactose solution for 5 min at 37°C and 2 times washing with the same solution [55].

### Sulfation of StxB sulf-2 and Ricin sulf-1/-2

StxB sulf-2 and ricin sulf-1 was prepared and purified as described previously [26], [37]. SiRNA-treated cells were washed twice with sulfate-free MEM containing L-glutamine and starved for 3 h at 37°C in sulfate-free medium, complemented with 0.2 mCi/ml Na_2_^35^SO_4_. StxB sulf-2, ricin sulf-1, or ricin sulf-2 was added and the incubation was continued for 1 h for StxB sulf-2, 2 h for ricin sulf-1 or 2–3 h for ricin sulf-2. StxB sulf-2 treated cells were washed once with PBS before lysis, whereas ricin sulf-1 or -2 treated cells were washed twice with 0.1 M lactose to remove surface-bound ricin and once with PBS before lysis with 0.1M NaCl, 10 mM Na_2_HPO_4_ (pH 7.4), 1 mM EDTA, 1% Triton X-100, Complete protease inhibitor (Roche Diagnostics, Mannheim, Germany) and 60 mM Octyl β–D-glucopyranoside (Sigma, St. Louis, USA). Lysates were centrifuged for 10 min at 8000 rpm and immunoprecipitated overnight at 4°C using protein A sepharose beads (GE Healthcare, Piscataway, NJ, USA), with either anti-Stx or anti-ricin antibodies adsorbed. Afterwards, the beads were washed twice with PBS containing 0.35% Triton X-100, resuspended in sample buffer and boiled for 5 min. The samples were separated by SDS-PAGE [56] and blotted onto ImmobilonP PVDF membrane (Millipore, Billerica, MA, USA). Autoradiography of the membranes was performed followed by densitometrical analysis using Quantity One® 1-D Analysis Software (Bio-Rad Laboratories Inc, Hercules, CA, USA). Proteins in the supernatant were precipitated using 5% trichloroacetic acid (TCA), dissolved in 0.1 M KOH, and analyzed for associated radioactivity to determine the total amount of sulfated proteins.

### Mannosylation of Ricin sulf-2

A modified ricin A subunit containing a tyrosine sulfation site and three partially overlapping *N*-glycosylation sites in the carboxyl terminus was produced, purified and reconstituted with ricin B chain to form ricin sulf-2 as previously described [37]. For the mannosylation of ricin sulf-2, cells were washed twice in glucose-free DMEM (Invitrogen) supplemented with 1.8 µl/ml D-(+)-glucose solution (10%) (Sigma-Aldrich) and starved in the presence of 0.1 mCi/ml D-[2-^3^H(N)]-mannose for 3 h at 5% CO_2_ and 37°C. After 3 h of incubation, ricin sulf-2 was added and the incubation continued for another 3 h. Subsequently, the cells were treated following the sulfation protocol described above for ricin sulf-1 and -2. For mannosylation experiments in the presence of Brefeldin A, cells were treated with or without 1 µg/ml Brefeldin A (BFA) for 30 min, followed by 3 h of incubation with ricin sulf-2 in presence of [^3^H]mannose.

### Determination of Cytotoxicity

The cytotoxicity of Stx and ricin was determined by the 50% reduction of protein biosynthesis in toxin treated cells. Therefore, cells grown in 24-well plates, transfected with different siRNA oligos, were washed twice in leucine-free Hepes medium, and increasing concentrations of the toxins were added. The incubation was continued for 1.5 h (Stx) or 2 h (ricin), then the medium was replaced with leucine-free Hepes medium containing 2 µCi/ml [^3^H]leucine, and the cells were further incubated for 20 min. The proteins were precipitated with 5% TCA, washed once in 5% TCA, and then dissolved in 0.1 M KOH. The incorporation of radioactively labeled leucine was quantified.

### Confocal Fluorescence Microscopy and Time-Lapse Microscopy

Cells were grown on glass coverslips and transfected with siRNA oligonucleotides targeting flotillin-1 or flotillin-2 and incubated for 3 days. For the experiments cells were incubated with 200 ng/ml of Stx or 1 µg/ml of ricin for the times indicated in the Figure legends at 37°C. Subsequently, the cells were fixed with a 10% formalin solution (Sigma-Aldrich), permeabilized with 0.1% Triton X-100/PBS, and immunostained with appropriate antibodies. Fluorophore-labeled secondary antibodies were obtained from Jackson ImmunoResearch Laboratories (West Grove, PA, USA). DRAQ5 (Alexis Biochemicals, San Diego, CA, USA) was used to stain the nuclei. The cells were mounted in Mowiol or Prolong®Gold (Molecular Probes, Eugene, OR, USA) and examined with a laser scanning confocal microscope LSM 510 META (Carl Zeiss, Jena, Germany). Images were prepared and analyzed with the LSM Image Browser software (Carl Zeiss) or ImageJ software. For time-lapse microscopy HeLa cells were transfected for 24 h with flotillin-1-GFP or flotillin-2-GFP. The time-lapse experiments were done by using a Nikon BioStation IM (Nikon Instruments Inc., Melville, NY, USA) and images were analyzed with ImageJ software.

### Statistics

For reproducibility all experiments were performed independently at least twice. Values of 3 or more parallels were given as mean ± standard deviation (SD). A *P* value of 0.05 or less was considered to be statistically significant and determined by the Student's *t*-test, ANOVA test or Mann-Whitney U-test.

## Supporting Information

Figure S1 Endosomal localization of flotillins and StxB sulf-2 in HeLa rab5 Q79L mutant cells. HeLa cells were seeded on glass coverslips and after 24 h they were treated for 30 min with 1 µg/ml StxB sulf-2, fixed, permeabilized, and labeled for Stx (green) and flotillin-1 or -2 antibodies (red). Pictures were analyzed by using Zeiss LSM Image Browser. Bars: 10 µm.
(0.42 MB EPS)

Figure S2 Mannosylation of ricin is inhibited in the presence of Brefeldin A. HeLa cells were transfected with 25 nM of the indicated siRNA oligos 3 d prior to the experiment. The cells were treated with or without 1 µg/ml Brefeldin A (BFA) for 30 min, followed by 3 h of incubation with ricin sulf-2 in presence of [^3^H]mannose. The cells were then lysed and ricin immunoprecipitated. The amount of incorporated [^3^H]mannose was determined by SDS-PAGE and autoradiography. The total protein mannosylation and ricin sulf-2 mannosylation was quantified and plotted + SD.
(0.29 MB EPS)
